# Community engagement in the prevention and control of COVID-19: Insights from Vietnam

**DOI:** 10.1371/journal.pone.0254432

**Published:** 2021-09-08

**Authors:** Bui Thi Thu Ha, La Ngoc Quang, Pham Quoc Thanh, Duong Minh Duc, Tolib Mirzoev, Thi My Anh Bui

**Affiliations:** 1 Hanoi University of Public Health, Hanoi, Vietnam; 2 Department of Global Health and Development, London School of Hygiene and Tropical Medicine, London, United Kingdom; Quaid-i-Azam University, PAKISTAN

## Abstract

**Introduction:**

Community engagement (CE) is an effective public health strategy for improving health outcomes. There is limited published knowledge about effective approaches to CE in ensuring effective responses to COVID-19 throughout lockdowns, travel restrictions and social distancing. In this paper, we contribute to bridging this gap by highlighting experience of CE in Vietnam, specifically focusing on migrant workers in Vietnam.

**Methods:**

A cross-sectional qualitative study design was used with qualitative data collection was carried out during August-October 2020. Two districts were purposefully selected from two large industrial zones. Data was collected using in-depth interviews (n = 36) with individuals and households, migrants and owners of dormitories, industrial zone factory representatives, community representatives and health authorities. Data was analyzed using thematic analysis approach. The study received ethics approval from the Hanoi University Institutional Review Board.

**Results:**

The government’s response to COVID-19 was spearheaded by the multi*-sectoral National Steering Committee for the Prevention and Control of COVID-19*, *chaired by the Vice Prime Minister and comprised different members from 23 ministries*. *This structure was replicated throughout the province and local levels and all public and private organizations*. Different activities were carried out by local communities, following four key principles of infection control: early detection, isolation, quarantine and hospitalization. We found three key determinants of engagement of migrant workers with COVID-19 prevention and control: availability of resources, appropriate capacity strengthening, transparent and continuous communication and a sense of trust in government legitimacy.

**Discussion and conclusion:**

Our results support the current literature on CE in infection control which highlights the importance of context and suggests that future CE should consider five key components: multi-sectoral collaboration with a whole-of-community approach to strengthen governance structures with context-specific partnerships; mobilization of resources and decentralization of decision making to encourage self-reliance and building of local capacity; capacity building through training and supervision to local institutions; transparent and clear communication of health risks and sensitization of local communities to improve compliance and foster trust in the government measures; and understanding the urgent needs ensuring of social security and engaging all parts of the community, specifically the vulnerable groups.

## Introduction

On January 30, 2020, the World Health Organization (WHO) declared the COVID-19 outbreak a public health emergency [1] and then on March 11, 2020 a Pandemic [2]. The pandemic is having a profound impact on global order and economy, and there are on-going concerns about health and well-being of people particularly the poor, vulnerable and disadvantaged [3–5].

Typically, responses to public health emergencies involve top-down actions by governments and public health agencies following bio-security approaches, and with little involvement of and consultation with the public [3, 6]. However, community engagement (CE) is an effective public health strategy for improving health outcomes and ensuring social support for the disadvantaged groups [7]. CE is defined as “*a process of developing relationships that enable of a community and organizations to work together to address health-related issues and promote well-being to achieve positive health impact and outcomes*” [8]. In low- and middle-income countries, CE has been a critical enabler of effective responses to controlling communicable diseases [9]. CE was effective in responding to the 2014 Ebola outbreaks in the contexts of a weak health system in Sierra Leone where community response teams were instrumental in interrupting the local transmission through contact tracing, house-to-house visits, health facility and community reporting [10, 11]. CE also contributed to significant decreases in child deaths due to malaria in Ethiopia, and reduction of HIV prevalence among sex workers, youth and general population [9, 12].

The term community encompasses all people, living in the same geographical area and sharing the same problems and resources, who know each another and often have a feeling of togetherness [13]. This includes community leaders (governing, traditional, and religious), community groups or networks or committees, health management committees, individuals and key stakeholders (students, women, elderly, youth and other vulnerable) [14, 15]. In response to emergencies, the whole community approach is recommended to engage the full capacity of all sectors, including profit and nonprofit, businesses, organizations, and the general public [6]. The six main themes of whole community approach are: understanding community complexity, recognizing community capabilities and needs, fostering relationships with communities, building and maintaining partnerships, empowering local action, and leveraging and strengthening social infrastructure, networks and assets [16].

Common approaches for CE include social mobilization, community participation, collaboration, community action empowerment [7, 17], typically throughout the design and delivery of interventions [9, 15]. Specific mechanisms for CE include sensitization and awareness raising, consultation, capacity building through training and supervision, developing social cohesion (building networking and trust), and strengthening links with health systems [15]. In relation to COVID-19, the main CE activities include effective communication for social and behavioral change, risk communication, surveillance and contract tracing [15].

The first case of COVID-19 in Vietnam, an Asian country where containment of COVID-19 so far has been relatively successful compared with countries in Europe and the Americas [18, 19], was declared on January 23, 2020. By November 30, 2020, Vietnam had over 1400 cases with 35 deaths, and in January 2021 the country is in the second wave of COVID-19 transmissions. Vietnam’s strict containment measures and integration of resources from multiple sectors have significantly reduced the spread of the pandemic in the country [19]. However, the need to engage vulnerable groups had been highlighted as an important consideration in responses to public health emergencies [20]. Migrant workers in Vietnam face a particular danger of having their livelihoods destroyed due to insecurity of work and income, and limited or no access to social security [21–23]. In Vietnam, migrants are categorised as those living, either independently or with relatives, outside their regular residence for over one month and who have temporary household registration books, or “tam tru” in Vietnamese. Most migrants in Vietnam are rural-to-urban and female [24] and often live in dormitories within or nearby industrial zones.

There is limited published knowledge about effective approaches to CE in ensuring compliance of vulnerable groups such as rural migrants, with strict government policies on controlling COVID-19 through lockdowns, travel restrictions and social distancing. In this paper, we contribute to bridging this gap by highlighting experience of CE in Vietnam, specifically focusing on migrant workers in Vietnam. This paper was guided by the following research question: what approaches and mechanisms of community engagement were applied in prevention and control of COVID-19, and what future lessons can be learned from these experiences? This paper should be of interest to academics and researchers who are interested in deepening their understanding of CE and to national and international decision-makers who may be interested in exploring approaches and mechanisms for effective CE within contexts of a dominant public sector.

## Materials and methods

A cross-sectional study design with qualitative data collection was carried out during August-October 2020. During this time the latest event related to Da Nang community outbreak was successfully controlled. Also, the Vietnam Ministry of Health, with support from WHO, had started communication activities supporting the “safe coexistence with COVID-19” initiative. To further support this, a long-term online campaign titled, “Normalize the new normal” had be jointly launched by United Nations organizations and other international organizations. Therefore, the current situation could be feasibility condition for conducting the study.

Two districts with large industrial zones (Que Vo and Phuc Son) were selected, from two provinces (Bac Ninh and Ninh Binh). These were purposefully selected, because industrial zones contain high proportions of migrant workers, and to allow comparisons across the urban (Phuc Son—Ninh Binh) and rural (Que Vo—Bac Ninh) settings.

The data was collected from 36 participants, including migrants and their households, and dormitories, industrial zone factory representatives, community representatives and health authorities (Table 1). In-depth interviews (IDIs) were conducted with purposefully-identified key informants to understand their experiences in CE in addressing the COVID-19 epidemic, specifically focusing on engagements of migrant workers. The interview guide consisted of tailored questions for different stakeholders, covering: stakeholder roles in COVID-19 prevention and control and how they were impacted by COVID-19; their understanding of CE during COVID-19 prevention and control, how they were implemented and what facilitators and barriers were encountered; what support was received and from whom as part of CE, including measures to facilitate compliance with relevant regulations. Experienced qualitative researchers conducted the IDIs, and explored the key approaches and mechanisms of the CE.

**Table 1:** Study participants.

Participants	Total (n = 36)	Female	Male	Age range	Position
**Individual & household**					
Migrant workers	4	3	1	26–37	Workers
Co-workers/ people sharing the same residence	4	4	0	24–37	Workers
Landlords of dormitories	4	0	4	51–67	NA
**Industrial zones (factories)**					
Health staff and labor union in the factories	4	2	2	35–40	Head
Managers in factories	3	1	2	32–45	Manager
Leader of industrial zones	2	1	1	39–49	Management board
**Community representatives**					
Leader of Commune Health Center (CHC)	2	1	1	47–48	Head
• Local Commune People Committee (CPC) • Police • Village leader • Mass organization (women or youth union)	7	2	5	32–53	Head of each agency
**Health authorities**					
Leader of Provincial Department of Health in charge of COVID-19	2	1	1	44–55	Vice director
Leader of Center of Disease Control in charge of COVID-19	2	1	1	39–46	Vice director
Leader of District Health Center in charge of COVID-19	2	0	2	45–54	Director

Review of several documents related to COVID-19 was also conducted to understand the current regulatory environment, from different level: governmental, ministerial and local.

All interviews were audio-recorded following due informed consent and transcribed verbatim. The IDI transcripts were coded following the approaches and mechanisms of CE by experienced qualitative researchers. The approaches included the collaboration and community empowerment. The mechanisms referred to mobilization of resources, capacity building, risk communication, engagement and leadership. The results were extracted and mapped based on the relevant codes. Data was analyzed using thematic analysis approach in Vietnamese and the codes and study report were written in English. Selective illustrative anonymized quotes from Vietnamese transcripts were translated verbatim into English for reporting.

The study received ethics approval from the Hanoi University Institutional Review Board (Approval number No.281/2020/HDDD). All primary data collection was conducted following obtaining of written informed consent from each participant and the reporting of results protected the participants’ identities.

## Results

**Fig 1:**
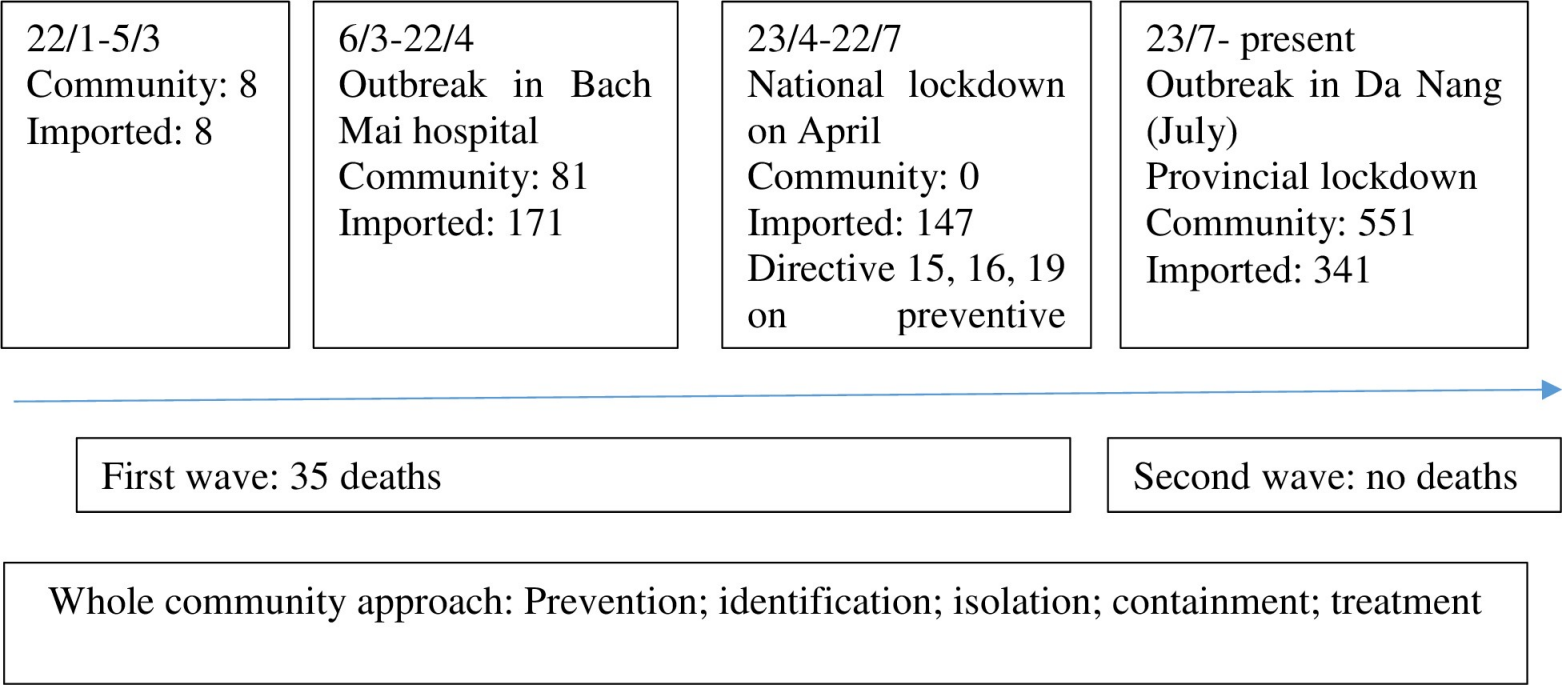
Timeline of COVID-19 in Vietnam by November 30, 2020.

Fig 1 presents the timeline of COVID-19 prevention and control in Vietnam. By November 30, 2020, there were 1331 cases and 35 deaths; including 674 new cases imported from abroad and 657 new cases in community [25]. Two outbreaks were reported in Bach Mai hospital (Hanoi) and Da Nang city.

Depending on level of outbreaks, different instructions were enforced by the government. On March 27, 2020, after the outbreak in Bach Mai hospital, the Government introduced strict limits on traveling and crowd sizes to less than 20 people [26]. On March 31, 2020 the national 4-week lockdown was introduced [27]. In July, 2020 the regional lockdown was set up for Da Nang city and neighborhood provinces following the outbreak in Da Nang city [28]. After lockdown lifted, the Government issued the Directive 19 with focus on new normal, with double mission on preventing and controlling of COVID-19 while maintaining economic activity [29]. The country is progressing with domestic recovery with retail sales and industrial production rebounding from low during the lockdown, while maintaining the prevention and control of COVID-19 [30].

We now present the themes that relate most strongly to approaches and mechanisms of community engagement. The summaries of themes were presented in Table 2.

**Table 2:** Themes identified from study.

Themes	Empirical data	Responses from key informants
Multi-sectoral collaboration through committee on COVID-19 prevention and control	• Committees on prevention and control of COVID-19 were established at all levels and agencies with involvement of different sectors • The roles of sectors were set according to their existing tasks and function • Strong leadership from Party and Government at all levels	• Community representatives • Health authorities • Leaders and managers in industrial zones and factories • Health staff and labor union in factories
Community empowerment in the prevention and control of COVID-19	• Decentralization of tasks to local level: every level should be responsible for the prevention and control of COVID-19 at their level with self-reliance principles • New partners emerged in contact tracing (police and youth union; women union) • New structures created in contract tracing (community COVID-19 supervision group)	• Community representatives • Health authorities • Industrial zones leaders • Managers in factories
Engagement migrant workers in COVID-19 prevention and control	• Compliance with regulation on prevention and control of COVID-19 at workplace and households (wearing masks, washing hand by sanitizers, measuring temperature) • Vulnerable groups (migrant workers) received different supports from different stakeholder such as social benefit package from government, company supports (allowance, in-kind supports), local organization (in-kind supports), landlords (in-kind supports). • Very difficult in getting social benefit package • Poor compliance after lockdown lifted (wearing masks, measuring temperature, washing hand by sanitizers)	• Community representatives • Migrant workers • Landlord
Mobilization of resources to support people, including migrant workers	• Willingness to contribute the resources (financial or in-kind) by different people and groups. • Difficulties in receiving social benefit package due to complicated procedures	• Community representatives • Migrant workers • Landlords
Capacity building through training and supervision by relevant stakeholders	• Training on prevention and control measures was provided by health sector to different agencies and institutions • Support supervision was organized to ensure the compliance with regulation in different places • Difficulties on compliance with keeping distance of 2 m and solutions implemented to comply with regulation in factories. • People aware of the severity of diseases and the needs for prevention and control of COVID-19 • Health sector was the technical lead and supported capacity building, but lacked resources and precautions after violation in CDC in Hanoi	• Health authorities • Managers in factory • Leaders in industrial zones
Risk communication on COVID-19	• Reliable and updated information on COVID-19 was broadcasted through different official media channels on daily basis • Local communication was activated through social media platform • Lack of clarity of health messages in early period • Unreliable news still existed and sanctions were implemented	• Health authorities • Community representatives • Industrial zones • Migrant workers and landlords

### Multi-sectoral collaboration through COVID-19 committees

Acknowledging the severity of pandemic, the National Steering Committee for the Prevention and Control of COVID-19 was established early, on January 30, 2020. The committee was led by the Vice Prime Minister and comprised different members from 23 ministries and sectors [19], and has been responsible for coordination of tasks, mobilization of resources, and providing information and communication to the public [19].

Each sector had to mobilize resources for the tasks that were relevant to their functions and capacities [19]. For example, the Ministry of Public Security is working closely with local authorities and health sector in tracing cases and at-risk contacts. The Ministry of Finance is responsible for social benefit package to those affected by COVID-19. The Ministry of National Defense provided space for centralized isolation points for infected cases or people returning from overseas. The Ministry of Foreign Affairs and Ministry of Transportation has responsibility to close borders with neighboring countries. The Ministry of Education is responsible for closing schools when necessary and providing online teaching. Finally, the Ministry of Health is responsible for developing guidelines and activities related to prevention, control and treatment of COVID-19 cases. Although health sector played significant role, most of key informants, reported strong beliefs on strong leadership of Party and national Government towards victory of combating COVID-19.

*Our country doing very well towards COVID-19. People support and believe on this process. Government lead and health sector is doing technical works. Without this, we could not contain the diseases. We see the failure of other countries, lot of cases and deaths. (PHD manager)*

The national steering committees were also established at provincial, district and commune levels and at all public and private organizations and agencies. These committees comprised representatives of local authorities, health sector, police, civil society or mass organizations such as women union, youth union, fatherland front and veterans. The chairperson or vice chairperson of local people committees often chaired the committee on COVID-19 prevention and control. The local committees were responsible for: detection of at-risk cases; reporting to higher level for isolation; quarantine and treatment; and enforcement of preventive measures in their location (wearing face masks in public places, hand-washing and sanitizing, social distancing, temperature measurements, crowd size management); [31], and reflected the decentralization of decision-making to local level as per the Government’s Directive 19. The leaders of committee had full responsibility on COVID-19 prevention and control, underpinned by four principles: local leadership, local resources, local facilities, instrument and materials and local logistics [31]. The local committee members had to be continuously available, as reflected by one participant.

*In situation of any risk case was reported, the chairman or vice-chairman of steering committee for COVID-19 prevention in community must be present at health commune center, requested the rapid response team and all the members of steering committee for COVID-19 prevention (i.e. health staffs, polices…) to access and get information from the household, guide for household to isolate at home. Then transferred drugs, sprayed disinfectants in that area surrounding the household. (CPC representative)*

### Community empowerment in the prevention and control of COVID-19

Different activities were carried out by local communities, following four key principles of infection control: early detection, isolation, quarantine and hospitalization. At the local level, police involvement emerged in the early detection of at-risk cases. The home visits were organized with involvement of health workers, local mass organizations (youth union or women union) and the police. The police were also responsible for safety of accompanying people and ensuring compliance of local communities with isolation, quarantine or hospitalization and their involvement was highly appreciated:

*While going to households in community, the policeman used to accompany us. The policeman and the vice chairman of the steering committee of COVID-19 prevention and control must accompany with us. Even though he was very busy with work, he still must go. When the emergency situation occurred, they must go to make a report even at night, otherwise the person with at-risk may be moved to another place tomorrow. So they must go and do it immediately even at night (CHC manager).*

In the industrial zones and dormitories, where most workers are migrants, the local police paid visits to discuss with leaders and landlords on their plans to comply with COVID-19 prevention and control measures. Such an approach strengthened the channels for monitoring people arriving from abroad and from outbreak regions

*When the pandemic occurred, we need to strengthen the supervision on people with high risk. We have the principles of following each street corner and each person and those should be under control (Police Representative).*

Community COVID-19 supervision groups were set up in all communes and villages. Each group consisted of 2–3 volunteers. They could be village health workers or member of youth or women unions. Each group was responsible for supervision of 40–50 households through: sharing information and advocating for compliance with preventive measures; requesting households to measure temperature on daily basis and self-report any symptoms; reporting symptoms from any household members (such as fever, cough) to local authority and commune health centers; reporting to local authorities all at-risk cases i.e. coming from outbreak areas, no health reports, and non-compliance with preventive measures; and tracing COVID-19 cases in the local areas [32]. During COVID-19 outbreak in Da Nang in July, total of 14,434 groups were set up in 4 provinces (Da Nang, Quang Nam, Quang Ngai and Quang Tri).

Following the instruction of the National Steering Committee on COVID-19 Prevention and Control, everyone arriving from outbreaks areas (Bach Mai hospital in March 2020 and Da Nang city in July 2020) [28, 33] had to be identified by the COVID-19 community supervision groups and report to the local authority or health center. The response team was sent to their house to obtain the travel history and contact with at-risk cases. Then, the decision is made by health professionals on sending for testing, isolation, quarantine or hospitalization to ensure the early detection of at-risk cases and reduce the spread of disease in the communities.

*It was a difficult problem in large area where people could not spread information quickly, then the participation of community supervision group was in need and very important. For example, people in community knew that the household had children, husband or wife who came from somewhere else, might be from the outbreak area. Then, we had to access immediately and direct the rapid response team to meet the household to get information of their historical travels such as where did they come from, where did they pass by…So that we could update information. If they came from the outbreak areas, we requested them go to medical isolation according to the government guidelines. (CPC representative)*

### Engagement of migrant workers in COVID-19 prevention and control

An industrial zone leader (female, 39 years) reflected that due to COVID-19, 25 out of 60 companies had to reduce or stop the production lines, and some companies did not sign long-term contracts and only kept 10–20% of their workforce. As consequence, migrant workers had to face layoffs or reduced working hours and falling incomes. Some suffered from anxiety and stress and those aged 45–50 years suffered most because of low perceived chances of finding jobs after the pandemic.

However, they still complied with preventive measures at workplaces, public spaces and their dormitories. In the companies, all workers have to measure temperature at the gates on arrival, wear face masks, wash hands with sanitizers, and keep a distance of 1–2 meters from other people if possible, even in the canteens. Although some migrant workers might not have wanted to disclose their health status due to fear of losing job and income, they are requested by the companies to report to health unit if they visited an outbreak region or have cough, fever or other COVID-19 symptoms and all workers have to comply with regulation.

*In case if someone coming from outbreak region, or having some symptoms or high temperature, the companies will request to stay home, and go to health center/hospitals for check-up. The companies also ask people not to go to outbreak region. (Migrant worker)*

During the lockdown period, all migrant workers complied with regulation on prevention and control. They wore face masks in the public places. Some did not go back to their hometown due to fear of transmission to their families.

*I did not come home for 4–5 months because fear of having contracted COVID-19. In my hometown, we do not have the diseases. Here, the cases are available, so I afraid I could get disease to other people, so I did not come home. (Migrant worker)*

In the dormitories, migrant workers were not allowed by landlords to gather in meetings or banquets due to fear of transmission of COVID-19. However, after the national lockdown was lifted in April, the community compliance with COVID-19 prevention has weakened due to perceived low chance of contracting disease as people were returning to normal.

*However, in phrase II, their practices were not quite good, they seemed to ignore, did not wear face-masks or forget to wear face-masks even the factory gave face-masks to them. It was as my opinion and observation in community. And I am a member of women union, so I just gave them a gentle remind to wear face-mask or comply with the guidance of COVID-19 prevention and control (WU representative)*

Our analysis reveals three key determinants of engagement of migrant workers with COVID-19 prevention and control, including compliance with government measures: availability of resources, appropriate capacity strengthening, transparent and continuous communication and a sense of trust in government legitimacy. Each are set out next.

### Mobilization of resources to support people in need, including migrant workers

The COVID-19 prevention and control required multiple resources. Different organizations and individuals willingly to contribute resources. In Ninh Binh, following calls from local authorities, in some communes each household voluntarily contributed 5 USD for COVID-19 prevention and control. Some in-kind support also was reported such as donations of face masks, hand-washing liquid and disinfection material. The mobilization of resources was spearheaded by the civil society organizations, Women’s Union and Youth Union.

*We established the campaign group, each group had 3 people. Firstly, this group advocated the household to isolate and not to go away from home to home. The campaign group just gave information, then the household would support. There were organizations and individuals had brought the money to support for the activities of COVID-19 prevention and control (CPC representative).*

People working directly with COVID-19 appreciated the local support, though also respected the anonymity of local donors.

*There are some people who donate but do not want to share their personal information. Someone supported more than 1,000 face-masks, I told them that I want to share a good example but they did not want to share their personal information. We also support the COVID-19 commune committee, the total is nearly 8 million VND [about USD 346]. We donated in the voluntarily spirit, someone in community just come and brought their donations (WU representative)*

Vulnerable groups, including migrant workers, received support from different stakeholders. They got the in-kind support including rice, clothes, face-masks, and subsidies on costs of accommodation, electricity or water from companies or landlords as one participant reflected.

*This is very difficult because I cannot list all the rental houses. But according to some information, we know that there are 14 landlords have reduced the renting room for workers. For example, they reduced from 700–800,000 VND to 300–400,000 VND. Reduced by half. Other landlords, they offered one month free of charge for renting room during that peak of COVID-19. (YU representative)*

Although substantial support has been offered to people in need, complicated procedures for obtaining social benefits package were reported. The criteria listed for beneficiary such as who losses the job or temporarily layoffs were very difficult to get the proved evidence. In reality, only small number of people could get the support (16 000 people from 1 million applicants) [30].

*The commune local authorities also organized the meeting to announce the government support package. Most people were applying for the support at the beginning. However, very few (7 people out of 223) got the support due to very strict criteria for being beneficiary people (WU representative).*

### Capacity building through training and supervision

To facilitate compliance with preventive measures, training and support supervision were organized by the health sector to the local organizations and agencies. The provincial center of disease control (CDC) organized training for large industrial zones on compliance with preventive measures and health risk communication. The supervision also was organized by provincial CDC and district health center in collaboration with Department of Invalids, Labor and Welfare to support the local agencies to meet the guidelines of prevention and control. The communication channel was set up between the local agencies and health sector to update information 24/7 days on COVID-19 situation.

*My company is under the management of Ninh Binh provincial CDC and Thanh Son commune health center. During the pandemic, health workers came and guided to follow the instructions from the Ministry of Health on COVID-19 prevention and control measures. So that, the company could understand and comply with the national regulations on COVID-19 prevention and control. (Factory health worker)*

The provincial CDC is responsible for supervision in areas where transmission is more likely to be very high (industrial zones, dormitory of migrants, high schools). The district health center (DHC) is responsible for supervision of primary schools in the local district and factories with more than 50 people with regards to social distancing, mask-wearing, hand-washing and meal arrangements in the canteens. The CHCs are responsible for supervision of factories and dormitories with less than 50 people.

In compliance with preventive measures in the companies, some challenges identified were difficulty in ensuring distancing of 1–2 m between people in factories due to existing production lines. However, under guidance from the health sector, some factories have applied some intervention solutions such as re-arranged starts of working shifts (from at 7 am to 7 and 8 am) to have less people in the same time at the entrance gates and reduce the risk of transmission; re-arranged meal times and putting partitions between dining tables to ensure social distancing in the canteens, requirement for all to wash hands before and after meals and allowing up to 50% of passenger’s capacity in the travel buses during COVID-19.

### Timely and transparent communication

The Ministry of Communication and Information is responsible for providing updated and reliable daily information on COVID-19 through different official channels (TV, radio), social media platforms (Facebook/Zalo), and SMS/mobile phone. The contents are provided by the Ministry of Health and include the number of new cases, deaths and outbreak region if available. The communication also includes information on preventive measures such as handwashing, face masks wearing, physical distancing. A total of 10 billion text messages were sent to 53 million mobile phone users up to November 30, 2020 [34]. The government companies also developed Ncovi and Blue Zone apps for self- reporting symptoms and cases of contacts with confirmed cases.

Locally, updated information on COVID-19 were shared among members of local committee on COVID-19 prevention and control using social media (Zalo/Facebook) and the chairperson of local committee were asked to keep their mobile phones on at all times to handle situations if needed. In addition, updated information was also posted in local radio, billboards, leaflets, meetings of Youth Union and Women’s Union groups with local people, including the migrant workers and their landlords, health units and workers in the factories. The landlords also provided information to the tenants and emphasize the need to follow the preventive measures.

People valued the updated and reliable information on COVID-19 as it helped to understand the symptoms, transmission and required preventive measures, as exemplified in the two quotes below from a health manager and a migrant worker, respectively.

*In my opinion, it was quite effective and very good role. Regarding health education and communication, at first, we need to give information to people who were not aware of COVID-19 prevention. Giving them information day by day and gradually it could bring the changes of their knowledge, attitude and practice. As I could see that, I just turned on the phone to get a medical report via Blue Zone app and I could read the title or updated information in short. Even people who did not pay attention, they still could find it. It was very effective. (CHC manager)*

*Government doing very well. I watch TV news every day, where the updated information on new cases and deaths every day in the world. This information helped me to trust and follow the government action and guidance (Migrant worker)*

However, at the beginning, multiple communications on social distancing or lockdowns and numerous instructions on preventive measures from different stakeholders (Party, ministries, local authorities, unions) created confusion on local implementation. Different rumors were shared on Facebook, such as incorrect information on new cases or appropriateness of lockdown measures. Increasing prices for food (rice, meat) and face masks before the first national lockdown, not wearing face masks in public places, and not self-reporting health status were also reported by the participants. Commensurate government sanctions included different fines for violation of health behaviors [35, 36] and communications [37].

## Discussion

Vietnam is one of few countries that has successfully controlled the COVID-19 so far, with just over 1,400 cases and 35 deaths by November 30, 2020 [25]. Arguably, strong multi-sectoral coordination minimized the traditionally siloed approaches and supported innovation when flexibility was required [38]. CE has been a critical factor in containing the epidemic [19, 39], similarly to controlling communicable diseases such as malaria, HIV and Ebola [9, 11, 12, 40]. In response to COVID-19, the CE with a community approach, which was deemed appropriate for emergency situation management [6] was deployed in Vietnam under the strong leadership of the Communist Party and the Government of Vietnam [41, 42].

The CE approach in Vietnam in containing the COVID-19 has been consistent with the current literature [7, 9, 15, 17] and involved multi-sectoral collaboration and strong government leadership reflected in establishment of relevant structures such as COVID-19 committees, and increased engagement from vulnerable migrant workers following mobilization of resources, capacity building and clear and transparent communication. The multi-sectoral collaboration across the relevant sectors also contributed to strengthen governance structures and creating context-specific partnerships throughout national, province and local levels [10, 13].

The local COVID-19 committees, which comprised health sector, police and mass organizations, benefitted from the intersections between sectors, and therefore facilitated the implementation of appropriate essential public health measures to contain the virus spread in the communities [42], such as health education for the public [20, 43]. Besides the technical role of the health sector, the new roles of the police and defense was instrumental to ensure the public’s compliance [39]. The community COVID-19 supervision groups is the evidence of newly-created effective local partnerships [8] that followed guidance from the national steering committee, alighted with a motto ‘combating pandemic like fighting the enemy’ [19].

Our results highlight effective roles of community supervision groups in addressing the epidemiological functions in Vietnam. However, such an approach also raises questions over personal freedoms of individuals and their families, for example related to required daily reporting of temperatures and reporting of non-compliance. On the other hand, experience shows that some countries with high degrees of personal freedom such as US and UK, failed to control pandemic due to failures to accept scientific public health advice on compulsory mask wearing and social distancing and weaker national coordination [16]. On reflection, this is a delicate balance which echoes the dominance of bio-security approach to controlling the epidemics while ensuring the local voice and genuine community engagement rather than compliance due to potential punitive sanctions [3–5].

In fact, decentralized decision-making process by local leaders with full responsibility towards COVID-19 prevention and control was emphasized by the national government. Each stakeholder was responsible for mobilization of resources for carrying out tasks commensurate to their functions and capacities, following the WHO guidelines [8]. The decentralization of decision making with self-reliance principles was highlighted as a feasible and successful approach for national diseases control programs [13]. Similar approaches were also adopted by the Chinese government in COVID-19 prevention and control, which may reflect similar political systems which involve strong national leadership and centralized guidance [43] to ensure consistent responses across the different provinces and districts. This is an important characteristic of effective infection prevention and control [44], though may not always be feasible in contexts where central guidance may be questioned by the local authorities such as for example in the UK or the US [18].

One important characteristic of CE is the participation of multiple vulnerable individuals and groups (including migrant workers) in multi-faced activities around behavior changes to ensure compliance with preventive measures in the workplaces, household and community settings. These findings echo results of other studies [14, 45, 46], though again a distinctive feature of the Vietnamese context relates to the degree of government’s influence and strong regulatory framework based on strict enforcement and commensurate sanctions for non-compliance. However, our results also show that the identification and addressing of needs of vulnerable migrant workers can be effectively done through a comprehensive engagement and support from factories, civil society organizations and landlords to migrants [20].

The study showed strong community compliance with government measures on prevention and control of COVID-19 at all levels and groups, including willingness to mobilize and contribute resources (Table 2). These may reflect established trust in the current public and private institutions [20, 43], though may of course be a mere reflection of strong regulatory framework. Trust is a critical component of community engagement and if communities lack of trust they will likely disengage with their health systems [47–50] and guidance from public authorities. We found that perception of risks, which are determined by linkages with local communities, strongly influenced willingness to prepare for emergencies [51] and determined degree of community trust to, and ultimately compliance with, government’s preventive measures [20, 52]. Our results also suggest that establishing and sustaining community partnerships which are built on effective interactions and continuous communication, can be effective in building and sustaining trust [40, 47, 48, 53], ultimately contributing to community engagement. Therefore, strengthening relationship and trust should be recommended to ensure the sustainability of future public health intervention programs.

Another important mechanism for facilitating CE is capacity building through clear communication, targeted training and supervision to people in need [6, 7, 20, 45]. These were found to be instrumental in directing prevention and control strategies and facilitating the integration of these activities in routine work of all organization and agencies [13].

We found that clear, transparent and timely communication of health risks is a crucial element of CE, echoing findings from other countries [43, 54, 55] and the guidance from the WHO [56]. In addition to improving public awareness and mitigating the risky behaviors, this also contributed to improving and maintaining trust [52]. Our results highlight that communication of risks should be in a simple and easy to understand format, so communities can further share knowledge locally. Communication of health-related information can target multiple audiences and serve different purposes, such as promoting actions and behavior change and providing guidance for healthcare professionals. Specific modes of communication can vary depending on the infrastructure [20]. In Vietnam, different technological innovations were introduced which included Ncovi and Blue Zone apps for health self-reporting, also echoing similar experiences from elsewhere [15]. The spread of fake news also faced timely sanctions in Vietnam [35, 36], and while this represents an experience to share [43] appropriateness of strict measures will ultimately need to be cognizant of local cultural environments such as degree of freedoms of speech and expression.

Local communities are part of the complex socio-cultural and economic ecosystems, and have important and active roles in prevention and control of diseases [10]. Clear links between public health agencies and the population are essential to ensure health outcomes [38]. However, it is also important to recognize that community engagement also relates to different times and places, and therefore is a process rather than an outcome. Stronger CE may emerge as the context changes and as opportunities are presented and seized, such as the formation of local committees or encouragement for mobilization of resources by the civil society organizations. CE interventions can be effective in a wide range of contexts and using a variety of mechanisms. Public health initiatives should therefore aim to integrate community engagement into the design and implementation all health interventions, not just the infection control [7].

Based on our results, we highlight five key components of effective community engagements: (1) multi-sectoral collaboration with a whole-of-community approach to strengthen governance structures with context-specific partnerships; (2) mobilization of resources and decentralization of decision making to encourage self-reliance and building of local capacity; (3) capacity building through training and supervision to local institutions; (4) transparent and clear communication of health risks and sensitization of local communities to improve compliance and foster trust in the government measures and (5) understanding the urgent needs on social security and engaging community, specifically the vulnerable groups. These components are inter-related, for example transparent and clear communication and whole-of-community approach can facilitate engagements of vulnerable groups in understanding their urgent needs. It is therefore important to deploy a comprehensive and holistic approach to CE which should entail implementing multiple and mutually-reinforcing components. These components are informed by results from the context of Vietnam with one-party government and a clear chain of command from the national down to the village level, which facilitated cross-sectoral coordination and ensured consistent messages while stringently enforcing regulations. However, our results also echo the wider literature and the five components are arguably applicable to variety of contexts. We recognize, however, that operationalization of the above components should be cognizant of context-specificity, issues such as potential multi-party political environments with different degrees of scrutiny, availability of scientific evidence and culture of evidence-informed policymaking, varying freedoms of expression and different degrees of public trust in their governments.

### Study limitations

The study provinces were purposively sampled for practical and resource reasons. We believe this sample provided insights into what was happening across Vietnam, though given considerable variability in the pandemic, results may not be fully generalizable. This relatively small-scale study aimed explored approaches and mechanisms of CE in combating COVID-19, focusing specifically on rural migrants. More research is needed to investigate the impact of COVID-19 on other vulnerable groups.

## Conclusions

Community engagement is critical for effective public health interventions, including infection prevention and control. In this paper, we explored the role of community engagement in the prevention and control of COVID-19 in Vietnam, specifically focusing on rural migrants. Five key components of effective community engagements are proposed: multi-sectoral collaboration with a whole-of-community approach, mobilization of resources and decentralization of decision-making, capacity building through training and supervision, transparent and clear communication of health risks and engaging all parts of community. Application of these components should be cognizant of local political, economic and cultural environments of different countries.

## Supporting information

S1 File Qualitative tool-IDI guideline. (RAR)

S2 File Qualitative transcripts. (PDF)
