# Supplementary material for: Community engagement in the prevention and control of COVID-19: Insights from Vietnam
Source: PLoS One. 2021 Sep 8;16(9):e0254432. doi: 10.1371/journal.pone.0254432 (PMC8425553; doi:10.1371/journal.pone.0254432)
Supplement: S2 File — (PDF) [file pone.0254432.s002.pdf]

SUMMARY OF THE TRANSCRIPTS RELEVANT TO MANUSCRIPT OF COMMUNITY ENGAGEMENT ASSESSMENT IN COMBATING COVID-19 IN INDUSTRIAL ZONES IN VIETNAM AMONG MIGRANT WORKERS

IDI-1-PHD manager-Male-55 years old

….

Q: Anh có thể nói qua cho bọn em biết là trogn giai đoạn vừa qua thì mình có những biện pháp chính gì tại tỉnh mình trong việc phòng chống dịch covid-19?

A: Nhìn chung thời gian qua nước ta đã triển khai tốt công tác phòng chống dịch COVID-19. Người dân ủng hộ và tin tưởng vào quá trình này. Biện pháp thì bên chính quyền, nhận sự chỉ đạo của bên ban chỉ đạo trung ương, ban chỉ đạo của tỉnh triển khai đến các ban ngành đoàn thể, về các văn bản, công văn hướng dẫn công tác phòng chống dịch của trung ương, thủ tướng chính phủ và của Bộ Y tế triển khai các công tác về chuyên môn và kỹ thuật. Ban chỉ đạo quốc gia, một mặt nữa chuyên ngành của mình là Sở y tế, thực hiện dưới sự chỉ đạo của Bộ Y tế, những công tác phòng chống dịch và những văn bản liên quan. Mình tiếp nhận các văn bản chỉ đạo đó, triển khai đến các đơn vị tuyến dưới thực hiện. Bên cạnh các văn bản triển khai về phòng chống dịch thì có nhiều cái cần chỉ đạo nhắc nhở, mảng truyền thông thì phối hợp với Sở truyền thông triển khai truyền thông công tác phòng chống dịch cho người dân nắm được. Tập huấn chuyên môn cho CBYT rồi những cán bộ chuyên ngành khác phối hợp làm công tác phòng chống dịch, cấp phát tờ rơi các kiểu. Đấy là công tác phòng dịch, còn 1 cái mảng nữa là trực tiếp đi triển khai công tác phòng chống dịch, giám sát cộng đồng, giám sát ở các cơ sở y tế phát hiện những ca bệnh mới, đồng thời kiểm tra giám sát công tác thực hiện phòng chống dịch. Nếu không có các hoạt động này thì chúng ta không thể đạt được kết quả như hiện nay, trong khi các nước khác số ca mắc mới và tử vong rất nhiều. (***→ Our country doing very well towards COVID-19. People support and believe on this process. Government lead and health sector is doing technical works. Without this, we could not contain the diseases. We see the failure of other countries, lot of cases and deaths. (PHD manager)***

Q: Anh ơi thế ở giai đoạn các địa phương lác đác xuất hiện sau ngày 17/3 ở HN có 1 trường hợp sau đó có 1 chùm ca bệnh ở Bạch Mai, thế những người ở địa phương khác họ di chuyển đến đây thì mình có làm công tác giám sát, theo dõi hay như nào không?

A: Cũng có một thời gian mình làm cái chặn ở các cửa ngõ, kiểm tra thân nhiệt, kiểm tra giám sát, cũng có những thời kỳ đi về Bắc Ninh mà không có mục đích chính đáng thì cũng không cho về, phải có xác nhận đến có việc gì chẳng hạn. Thế nhưng cái đấy cũng triển khai trong thời gian ngắn thôi xong thôi.

Q: Thế còn những đối tượng là công nhân ngoại tỉnh thì mình có bất kì biện pháp gì vì họ có những di chuyển đi lại từ các tỉnh khác về đây?

A: Công nhân ngoại tỉnh trong thời gian quản lý chặt nhất ấy thì vẫn cho họ vào với điều kiện phải kiểm tra chặt chẽ, thứ nhất là cái công tác phòng chống dịch phải dãn cách, lúc bấy giờ cái xe 45 chỗ chỉ được 20 người, ví dụ như samsung họ triển khai cũng như các nơi khác, không được ngồi đông như bình thường, có nước rửa tay khô, có khẩu trang đeo, chính vì thế nên trường hợp ở Hạ Lôi do samsung làm tốt nên cháu ấy không lây cho ai, công nhân đông thế nhưng không lây cho ai, quản lý rất chặt, chỉ có mình nó bị thôi.

….

IDI-2-CPC representative-Male-46 years old

….

Q: Từ việc thành lập ban chỉ đạo, tổ chức triển khai các hoạt động thì anh thấy là mình đều có quy định phân công chức năng nhiệm vụ cho từng thành viên trong ban chỉ đạo đúng không ạ? Có quyết định thành lập ban chỉ đạo không ạ?

A: Có

Q: Trong đợt vừa qua mình không có trường hợp nào phải chuyển lên tuyến trên hay cách ly như thế đúng không ạ?

A: Trên địa bàn bọn anh không có người nhiễm. Nhưng cái gọi là nghi ngờ F2 thì có.

Q: Mình xử lý như thế nào ạ?

A: Khi có thông tin thì trưởng/phó ban chỉ đạo phải có mặt tại trạm, yêu cầu toàn bộ trực tiếp có những ai thì tất cả những nơi đó anh phải đến và yêu cầu y tế, công an, tổ phản ứng nhanh đến có mặt luôn với gia đình, phối kết hợp nắm thông tin và hướng dẫn gia đình cách ly tại nhà. Chuyển thuốc và đánh phòng, phun khử trùng tại khu vực đó… ***(→ In situation of any risk case was reported, the chairman or vice-chairman of steering committee for COVID-19 prevention in community must be present at health commune center, requested the rapid response team and all the members of steering committee for COVID-19 prevention (i.e. health staffs, polices…) to access and get information from the household, guide for household to isolate at home. Then transferred drugs, sprayed disinfectants in that area surrounding the household. (CPC representative)***

Q: Trong triển khai các hoạt động này, như anh chia sẻ thì sự phối hợp của các ban ngành rất là cần thiết. Trong quá trình triển khai thực hiện trong thời gian vừa rồi, anh đánh giá thế nào về sự phối hợp giữa các ban ngành trên địa bàn xã mình? Từ cá nhân anh đánh giá như thế nào về sự phối hợp giữa các ban ngành nó có thuận lợi hay khó khăn gì hay có điểm nào anh thấy nó cũng chưa làm được tốt lắm và nguyên nhân của cái việc đó là gì? Phối hợp này bao gồm cả phối hợp giữa các thành viên trong ban chỉ đạo trên địa bàn xã, cũng như là phối hợp với các cơ quan ban ngành ở tuyến huyện hoặc là tuyến tỉnh chẳng hạn.

A: Trong quá trình triển khai thực hiện, có sự phối hợp vào cuộc của các ban ngành đoàn thể là một trong những cái để đạt được hiệu quả cao, cần có sự đồng thuận phối hợp chặt chẽ của các ngành đoàn thể trong khối đại đoàn kết. Chúng ta thực hiện trong công tác tuyên truyền hay nắm bắt tình hình. Mình phải khẳng định việc chỉ đạo sát sao của lãnh đạo là một trong những cái có hiệu quả. Có những cái để làm căn cứ trong công tác lãnh đạo chỉ đạo. Trong những trường hợp cấp bách, công tác giải thích tuyên truyền, giao nhiệm vụ cụ thể cho từng thành viên trong ban chỉ đạo. Cũng như là sự phối kết hợp của các ngành đoàn thể khi nắm tình hình và khi có diễn

biến xảy ra trong thôn xóm thì bắt buộc những người đã được giao nhiệm vụ phải có trách nhiệm. Để rồi có sự phối hợp khi có thông tin, bọn anh có chỉ đạo là khi nhận được thông tin thì các đồng chí đang làm bất cứ nhiệm vụ gì phải thông báo về để có mặt, bất đắc dĩ không thể có mặt ngay được, đồng chí có thể cử đồng chí phó, đồng chí thường trực của các ngành đó có mặt để phối kết hợp thực hiện. Đó là một cái trong góc độ lãnh đạo chỉ đạo đơn vị thực hiện phải có văn bản,các văn bản có quy định cụ thể, trong góc độ chỉ đạo thực hiện nhiệm vụ thì có sự chỉ đạo chặt chẽ, sát sao. Chỉ đạo xong ta phải có kiểm điểm và nhắc nhở. Phải có sự chỉ đạo trực tiếp. Người dân phản ánh lên cái là mình phải có sự chỉ đạo bằng cái tiếp nhận chung rồi, nhưng trực tiếp bằng điện thoại, mình phải đến để yêu cầu những người đó. Đấy là một cái để thuận lợi thì phải có công tác đó. Thứ hai là sự phối hợp giữa ngành đoàn thể dưới thôn xóm, có sự vào cuộc chung. Để người ta hiểu được thì công tác chỉ đạo lãnh đạo phải chặt chẽ, các thông tin hướng dẫn, văn bản mình cung cấp đầy đủ để người ta nắm bắt được tình hình diễn biến của dịch – gọi là thông tin, người ta nắm được thông tin rồi người ta mới phối hợp được. Phối hợp có vấn đề gì sẽ có sự trao đổi trực tiếp ngay. Nắm bắt được và trao đổi ngay. Trong nội bộ các hoạt động phải liên tục và trao đổi nhanh nhất với nhau. Ba là có sự vào cuộc của cả nhân dân, trong đó tuyên truyền đến người dân để người dân nắm bắt được và có sự báo sự báo cáo. Có cái đang tồn tại là địa bàn rộng thì người ta nắm bắt chưa được nhanh thì phải có sự vào cuộc của người dân. Người dân phát hiện nhà này có người con, chồng, vợ đi ở nơi nọ nơi kia về.... không biết ở nơi nào nhưng nay thấy có mặt ở nhà chẳng hạn. Thì bọn anh phải phối kết hợp ngay, chỉ đạo luôn đội phản ứng nhanh xuống gặp gỡ gia đình lấy thông tin người này ở đâu về, qua vùng nào……Để mình có những cái cập nhật ngay. Đúng ở vùng dịch về hay đi qua vùng dịch thì mình yêu cầu phải có sự cách ly y tế tập trung. Những cái đó bọn anh thông tin ngay tức khắc từ dưới lên trên luôn, từ ban chỉ đạo xã thông tin kịp thời lên ban chỉ đạo thành phố cũng như tỉnh để phối hợp chúng ta làm luôn....***(→ It was a difficult problem in large area where people could not spread information quickly, then the participation of community supervision group was in need and very important. For example, people in community knew that the household had children, husband or wife who came from somewhere else, might be from the outbreak area. Then, we had to access immediately and direct the rapid response team to meet the household to get information of their historical travels such as where did they come from, where did they pass by…So that we could update information. If they came from the outbreak areas, we requested them go to medical isolation according to the government guidelines. (CPC representative)***

…..

IDI-1-CPC representative-Male-53 years old

Q: Ban chỉ đạo là mình bao nhiêu thành viên ạ?

A: Đầu tiên mình thành lập là 15 thành viên, thực sự là tình nguyện vào, người đưa họ vào họ không muốn nhưng người lại tình nguyện vào.

Q: Ai được đưa vào nhưng họ không muốn ạ?

A: Những người như ở hội phụ nữ rồi đoàn thanh niên những người dân, ban chỉ đạo thì có rồi nhưng có những người là tình nguyện đi vận động doanh nghiệp vận động đeo khẩu trang. Khi mà mình phát động nhân dân về quyên góp ủng hộ về vật chất hay tinh thần là người dân xúc tiến ngay, cái vận động ủng hộ covid so với các vận động khác như lũ lụt hay đồng bào khó khăn thì thời gian là ngắn hơn và số người ủng hộ nhiều hơn. Chỉ trong 2 ngày được gần 20 triệu vượt chỉ tiêu gần gấp 2 lần.

Q: Những nguồn đấy là từ đâu ạ.

A: Thành lập từng ban vận động đầu tiên là cách ly là không cho đi từng nhà khi mà đến mỗi tổ 3 người mỗi người vào từng nhà 1, thông tin cái là người ta ủng hộ luôn, có những tổ chức cá nhân mang tiền đến tận ủy ban ủng hộ luôn. Cái khó khăn là 1 số người công nhân đến ở ý thức hoạt động rất là kém nhất là những người thanh niên nhưng cũng không ảnh hưởng lắm, có những lúc đi ra đường khẩu trang không đeo nhắc nhở còn rút dao ra đâm sau đó bị phạt tù ngồi. cũng rất nghiêm. Đặc biệt chủ yếu là thanh niên nó chủ quan hơn.

Q: Bên địa phương mình có biện pháp hay công tác khắc phục gì không anh?

A: Cứ làm công tác tuyên truyền vận thôi, và sau chỉ đạo của huyện thì ban thành lập 2 tổ gác ở 2 đầu đường là những nơi công nhân họ đi đén đi làm đông nhất cũng là đo than nhiệt rồi thì yêu cầu đeo khẩu trang. Có những người tình nguyện đến đây cầm máy đo thân nhiệt, dù buổi sang có rất nhiều người đi lại. đông như thế nhưng chưa có đối tượng nào bị nhiễm… ***(→ We established the campaign group, each group had 3 people. Firstly, this group advocated the household to isolate and not to go away from home to home. The campaign group just gave information, then the household would support. There were organizations and individuals had brought the money to support for the activities of COVID-19 prevention and control (CPC representative).***

…..

IDI-2-CHC manager-Female-48 years old

….

Q: Thế trong cái quá trình triển khai các cái hoạt động, chị nhận xét thế nào?

A: Nói chung thì chị thấy ở địa phương làm rất là tốt, ở địa bàn của chị thì chị thấy ủy ban họ chỉ đạo là làm rất đồng bộ vì em đến đây hôm nay mà chị Bí Thư ở nhà thì em cũng nên gặp, họ nắm rất là chắc, từ các ban ngành đoàn thể thì họ đều có mặt trong cái ban chỉ đạo phân công nhiệm vụ rồi họ về họ phải triển khai cho các hội viên của họ, dù họ không tụ tập nhưng mà họ vẫn phải triển khai, họ triển khai trên cái cơ sở ấy thì họ phân họ sẽ tuyên truyền được cho rất nhiều người, nhiệm vụ nào họ cũng phải hoàn thành, giao nhiệm vụ là phải hoàn thành, các em Đảng chính quyền là phải thực hiện.

Q: Thế nhưng mà trong quá trình ấy thì chị thấy là cần rất nhiều người phải đi ví dụ như công an, như chị nói là đi đâu làm gì thì cũng cần công an phải đi cùng thì mình có đủ lực lượng công an để đi theo mình trong những đợt đó không? Có bị thiếu không ?

A: Đương nhiên họ thiếu thì họ phải chạy từ chỗ nọ sang, đây như em gọi thì chị kia đang vừa đi họp thì phải chạy về còn gì nữa. Họ điều hành thôi thì mình cũng phải tuân thủ giờ giấc, cũng phải linh hoạt, cũng có người đến ngay được, cũng có người phải đợi 5-10 phút, 1 tiếng chẳng hạn các anh ấy mới đến.

Q: Có bao giờ mà huy động mà không có người đi không?

A: Không, họ cũng muốn đến để họ nắm được tình hình về báo cáo nên họ cũng muốn đi với mình, mà hơn nữa là nói thật với em đi xuống cơ sở thì nhiều người họ cũng ương mà có công an thì mình làm việc dễ ngay còn không có công an là mình làm việc khó ngay

Q: Vâng, thì tất nhiên phải có công an nhưng mà ý em hỏi là có bao giờ bị thiếu không?

A: Riêng công an các anh ấy sắp xếp không bao giờ thiếu, đi ngay sau khu công nghiệp vậy không bao giờ thiếu, công an họ chỉ đi xe họ đến cái thì Baries họ mở ra ngay

Q: Thế còn Ví dụ như nếu mà có có cái trường hợp nào mà nhiễm xảy ra trên địa bàn hoặc là nghi ngờ gì đó thì các cái hoạt động xét nghiệm rồi là khám sàng lọc thì thực hiện như thế nào ạ?

A: Em ơi những cái trường hợp nghi đấy thì các chị lại phải kết hợp với công an với lại trưởng thôn bí thư ở địa bàn mình xuống tận nơi để xem… Thì bọn chị là xuống nhà dân, đi với các chị bao giờ cũng có công an chưa bao giờ không có công an với cái anh phó chủ tịch này bao giờ cũng phải đi, đấy bận công việc mấy ông ấy cũng đi đêm hôm ngủ đi. Gọi chị đi lập biên bản ban đêm cũng có nếu mà không ngày mai họ lại chuyển đi nơi khác, cho nên là phải đi ngay… ***(→ While going to households in community, the policeman used to accompany us. The policeman and the vice chairman of the steering committee of COVID-19 prevention and control must accompany with us. Even though he was very busy with work, he still must go. When the***

emergency situation occurred, they must go to make a report even at night, otherwise the person with at-risk may be moved to another place tomorrow. So they must go and do it immediately even at night (CHC manager).

…...

IDI-1-CHC manager-Male-47 years old

….

Q: Trong cái ban chỉ đạo này thì cái cập nhật thông tin ấy thông thường trong ban chỉ đạo là mình trao đổi thông tin với nhau bằng cách nào.

A: Trong lần đầu ấy chúng tôi cũng chỉ đạo sau đó kết nối trên zalo nhóm từ ban chỉ đạo phòng chống thì sẽ vào cái nhóm của phòng chống covid, tất cả văn bản chỉ đạo báo cáo thông tin báo chí là cập nhật hết vào đây. Do vậy tất cả mọi người từ thông báo của các xã khác đảm bảo thong tin luôn cập nhật.

Q: Ngoài zalo ra thì còn hình thức nào trao đổi nữa không ạ?

A: Nói chung có các công văn chỉ đạo bằng thông báo hay chỉ thị có các cái đấy thường xuyên và liên tục, có gì là chúng tôi cập nhật thông tin ngay. Có gì là trong ban chỉ đạo đều nắm được hết và sau đó là chúng tôi có các bài tuyên truyền.

Q: Tần suất truyền thông trong giai đoạn ấy có tăng lên không ạ?

A: Chúng tôi tăng đột biến.

Q: So với trước đây tăng lên như nào ạ?

A: Thực ra là y tế chúng tôi trước đây là phát thanh là thứ 2 với thứ 5 hàng tuần khi mà covid xảy ra thì 1 ngày 3 buổi tính ra có những lúc nó lên khá nhiều

Q: Truyền thông là do bên văn hóa xã thực hiện ạ?

A: Đối với chusg tôi thì có các lãnh đạo trong ban chỉ đạo thứ nhất là ban văn hóa, trưởng đoàn và trạm y tế. chúng tôi cung cấp thông tin và chúng tôi cũng viết bài sau đó văn hóa là chỉnh sửa câu từ sau đó phó chủ tịch duyệt đưa cho đagi truyền thanh đọc và gửi về cho các thôn.

Q: Ở thôn có loa hết ạ?

A: Có hết. 4 thôn đều có, các đồng chí đến sớm thì sẽ được nghe.

Q: Đánh giá 1 chút cái vai trò của cơ quan truyền thông cũng như là công nghệ thông tin trong giai đoạn dịch này thì anh thấy như thế nào ạ?

A: Tôi thấy là khá hiệu quả, vai trò rất tốt. đối với truyền thông giáo dục sức khỏe thì đầu tiên người ta chưa nhận thức được. phải đưa thông tin tới cho người ta, nay không nghe được thì mai ngày kia sẽ nghe được nó tạo thành 1 cái luồng sóng, mỗi hôm 1 ít. Tôi thấy là chỉ cần mở điện thoại ra thì có khai báo y tế có bluezon có các cái tit trên các trang ngắn thôi nhưng hay. Những người không để ý thì người ta mở ra sẽ thấy, rất hiệu quả. ***(→In my opinion, it was quite effective and very good role. Regarding health education and communication, at first, we need to give information to people who were not aware of COVID-19 prevention. Giving them information day by day and gradually it could bring the changes of their knowledge, attitude and practice. As I could see that, I just turned on the phone to get a medical report via Blue Zone app and I***

could read the title or updated information in short. Even people who did not pay attention, they still could find it. It was very effective. (CHC manager)

Q: Trong cái thời gian tiếp theo vì dịch hiện nay vẫn chưa kết thúc thì anh có đề xuất gì liên quan đến hoạt động để đảm bảo sự liên kết giữ acasc bên tham gia trong phòng chống dịch trong cộng đồng nói chung cũng như với khu công nghiệp các công nhân nhập cư để đảm bảo sự cam kết và tham gia phối hợp các ban ngành đoàn thể ở địa phương ấy.

A: Theo tôi thì cái gắn kết giữa các khu công ghiệp và địa phương ấy cũng rất quan trọng, khi gắn kết được mới quản lý theo dõi được các đối tượng.

….

IDI-2-Police Representative-Male-38 years old

….

Q: Trong cái hoạt động tham gia phòng chống COVID đã diễn ra tại đơn vị mình cơ sở mình thì anh có thể mô tả giúp 1 chút cái hoạt động hiện nay về phía công an của mình trong việc tham gia truyền thông cũng như là cung cấp thông tin phòng chống COVID 19 được diễn ra như thế nào?

A: Đối với lực lượng công an ấy thì được sự chỉ đạo xuyên suốt từ các cấp lãnh đạo cũng như là chính quyền địa phương thì trên thành phố cũng thành lập 1 ban chỉ đạo phòng chống COVID của lực lượng công an nói riêng, thì đối vứi cấp xã thì chính quyền địa phương cũng thành lập 1 ban chỉ đạo dop đồng chí chủ tịch UBND làm trưởng ban trên thành phố thì cái lực lượng công an của mình ấy thì đòng chí trưởng công an thành phố làm trưởng ban chỉ đạo phòng chống COVID. Thì xác định ấy cái việc phòng chống COVID là như chỉ đạo của chính phủ là chống dịch như chống giặc nên là tất cả các lực lượng công an đều dốc sức làm tròn cái chức trách nhiệm vụ được giao. Về cái lực lượng công an xã ở dưới này thì cũng là 1 thành phần trong ban chỉ đạo phòng chống COVID phối hợp với chính quyền địa phương đặc biệt là với ý tế của xã thì làm tốt công tác rà soát, nắm người nắm hộ, nắm từng người 1 người để n người đi người từ địa phương khác đến và người lưu trú để phát hiện ra những trường hợp mà những nguồn dịch từ những nơi khác đến và có những cái tham mưu cho ủy ban cũng như là y tế để mình có biện pháp cách ly phù hợp và đặc biệt là phối hợp với y tế trong quá trình cách ly. Còn trong cái giai đoạn mà thực hiện chỉ thị 15, 16 của chính phủ thì lực lượng dưới sự chỉ đạo của lãnh đạo công an thành phố là công an xã Ninh Phúc làm rất quyết liệt trong cái vấn đề là giãn cách xã hội. và cũng đã trực tiếp tham mưu cho UBND xã kiểm tra xử phạt 1 số hộ kinh doanh không chấp hành chỉ thị 15, 16 của chính phurvaf trong cái tháng cao điểm nhất tức là cái đợt COVID lần thứ nhất ấy thì cơ bản là công an xã làm rất tốt cái trò tham mưu và trực tiếp phòng chóng dịch COVID.

Q: Tập trung nhất là cái mảng truyền thông thì mình có tham gia cùng với bên y tế không hoặc là các bên liên quan khác không.

A: Trong cái vấn đề về cái truyền thông là cái vấn đề rất là cốt lõi thứ nhất là dưới sự chỉ đạo của ban lãng đạo công an thành phố chúng tôi đã thành lập những trang fanpage trên mạng xã hội thành phong trào cái thứ 2 là trên trang nhóm nội bộ của xã và đặc biệt là đài truyền thanh của xã thì thường xuyên tuyên truyền về cái nguy cơ và nguy hiểm của cái dịch COVID 19 đặc biệt là tuyên truyền về cái chấp hành chủ trương đường lối chính sách theo quy định của pháp luật về phòng dịch thì cơ bản người dân nhận thức rất cao về cái vấn đề chống dịch COVID đặc biệt là chúng tôi còn tăng cường bên các khu công nghiệp chúng tôi trực tiếp có các cán bộ đi vào tuyên truyền bằng các văn bản cũng như là trực tiếp gặp lãnh đạo để tuyên truyền về phòng chống dịch COVID phối hợp với y tế nữa cơ bản là tất cả đều nêu cao cái ý thức đặc biệt là những doanh

nghiệp có vốn đầu tư nước ngoài , người ta đều nêu cao ý thức phòng chống dịch thì về cơ bản là cái công tác tuyên truyền cũng làm rất tốt, người dân thì người ta chủ động trong công tác phòng chống dịch.

Q: Liên quan đến sàng lọc và theo dõi các bệnh nhân ví dụ như là f1, f2 hiện nay mình đang thực hiện trobg đoạn theo dõi vừa rồi mình sàng lọc và cách ly các bệnh nhân này như thế nào?.

A: Cái vấn đề sàng lọc và cách ly là chuyên môn của y tế, khi mà y tế người ta có những cái biệp pháp tức là công an phối hợp cùng với y tế để phát hiện ra những nguồn dịch về, 1 là nguồi y tế 2 là từ nguồi công an để phát hiện thì sau khi sàng lọc y tế người ta sẽ có những cái theo cái chuyên ngành người ta thì người ta sẽ đưa vào 1 là cách ly tập trung 2 là cách ly tại cộng đồng thế thì trong quá trình phối hợp lực lượng công an cũng đóng vai trò đảm bảo an ninh trật tự và đảm bảo thực hiện chỉ thị của chính phủ trong công tác thực hiện phòng chống dịch bệnh, phối hợp với lực lượng y tế làm tốt vai trò. Thế còn trực tiếp giám sát rồi kiểm trai thực hiện đo thân nhiệt tức là chuyên môn của y tế thì người ta thực hiện chuyên môn của người ta còn công an thì thực hiện chuyên môn của công an trong đó thì chủ tịch cũng giao cho lực lượng công an ví dụ như là cách ly tại nhà cũng giao cho lực lượng công an cùng với y tế giám sát xem là có việc nguời ta rời khỏi nhà không, lực lượng công an cũng phối hợp chặt chẽ với y tế làm tốt cái việc đấy.

Q: Có khó khăn gì liên quan đến cơ sở vật chất, trang thiết bị hay công cụ hỗ trợ từ phía bên mình không?

A: Đối với lực lượng công an thì hầu hết là cũng được sự quan tâm của công an cấp trên thì cũng nói chung là cơ bản trong giai đoạn chống dịch thì về cái nhiệm vụ của lực lượng công an chính quy về xã thì nó cũng là cái nghiệm vụ thường xuyên thôi bởi vì thực tế chúng tôi không phải cứ có dịch mới giám sát địa bàn mà hầu hết đây là công tác thường xuyên liên tục của chúng tôi nên là đối với cái vấn đề khi mà có dịch bệnh xảy ra chúng tôi chỉ cần tăng cường thêm 1 chút nữa thôi về cái công tác bám sát dân nôm la như mình nói là bám từng nhà rà từng người ấy thì chúng tôi sẽ làm tốt công tác ấy ....(***→ When the pandemic occurred, we need to strengthen the supervision on people with high risk. We have the principles of following each street corner and each person and those should be under control (Police Representative).***

….

IDI-2- Migrant workers-Male-31 years old

….

Q: Trong giai đoạn covid thì khi mà mình tổ chức ăn uống cho công nhân thì thực hiện như thế nào?

A: Trong những thời cao điểm thì công ti sẽ chia theo múi giờ tức là ăn với cái số lượng công nhân viên đi cùng 1 lúc hạn chế tiếp xúc nhiều.

Q: Mình chia làm mấy múi anh nhỉ?

A: Bữa trưa chia thành 4 múi.

Q: Mấy giờ ạ?

A: Từ 11h15-13h.

Q: Các anh chị công nhân có phản hồi gì về cái việc chia giờ này không ạ?

A: Thì họ cũng không có phản hồi gì cả, tại vì đấy là cái quy định luân chuyển có sự thích nghi để thay đổi, nó không có vấn đề gì cả.

Q: Cái cao điểm cách ly thì mình có hoạt động gì khác không?

A: Trong đoạn đấy thì công ti yêu cầu các đối tượng đi từ vùng dịch về thì tự cách ly theo quy định của bộ y tế, ngoài ra thì đối với tất cả các cái trường hợp mà đến từ vùng dịch thì sẽ cấm hoàn toàn không được tiếp xúc trong công ti, ngoài ra thì tất cả các thành viên công nhân viên đều phải khai báo y tế trong quá trình làm việc và cập nhật thường xuyên thong tin các ca bệnh và xem là những đối tượng F1, F2 có trực thuộc làm việc tại công ti, sẽ khuyến cáo và yêu cầu cách ly luôn. ***(→ In case if someone coming from outbreak region, or having some symptoms or high temperature, the companies will request to stay home, and go to health center/hospitals for check-up. The companies also ask people not to go to outbreak region. (Migrant worker)***

Q: Mình có thực hiện khai báo y tế ở công ti mình không?

A: Khai báo y tế ở công ti thì có.

Q: Cái tần suất thực hiện như nào?

A: Tần suất thực hiện thì chỉ áp dụng với những trường hợp đến từ vùng nguy cơ sẽ tiến hành khai báo y tế và bắt buộc theo dõi.

Q: Trong quá trình đơn vị mình triển khai chống covid thì khó khăn mình gặp phải phía công ti cũng như cá nhân mình như thế nào anh?

A: Hiện tại cái vấn đề triển khai chống covid không khó khăn gì cả bởi vì cái dịch bệnh là toàn cầu, cái ý thức của cán bộ công nhân viên lao động được nâng cao rất nhiều, nên các vấn đề triển khai các biện pháp phòng chống thì hoàn toàn là ý thức của người lao động.

….

FGD-1-Migrant workers-Female-26 years old_01

….

Q: Khi mà có dịch Covid xảy ra ấy thì bọn em thấy là công việc của mình có bị ảnh hưởng hoặc thay đổi gì không?

A1: Về cơ bản thì cũng không có thay đổi nhiều lắm ạ, chỉ là trong trường hợp ví dụ như nếu có đi qua vùng dịch mà thân nhiệt bất thường thì công ty sẽ có những chính sách yêu cầu đầu tiên là ở nhà cách ly trước và theo dõi, và nếu như trường hợp sốt cao quá thì có thể đến bệnh viện để khám xem là tình hình dịch như thế nào. Vâng, cơ bản thì nó cũng không có ảnh hưởng nhiều lắm.

Q: Giờ làm rồi ca làm việc của bọn em có bị thay đổi không?

A1: Không.

A2: Bên em có giờ hành chính nên cơ bản cứ làm 8h/ ngày thôi ạ.

Q: Thế còn ví dụ như chắc là ca làm không thay đổi thì lương mình cũng không bị thay đổi gì đúng không?

A1: Dạ vâng.

A2: Thay đổi là ví dụ như là phải đeo khẩu trang thôi bởi vì đây là bắt buộc.

Q: Thế đợt dịch vừa rồi thì có lúc nào đi về nhà được không?

A1: Không.

A2: Em khoảng 4-5 tháng rồi chưa về, cũng sợ lây cơ. Bởi vì chỗ em ấy thì chưa có cái kiểu là người nào bị mắc bệnh ấy, còn ở đây thì em sợ ấy, thi thoảng không về nhà sợ là gây cho… có ảnh hưởng gì đấy nên là thôi không về nữa. ***(→ I did not come home for 4-5 months because fear of having contracted COVID-19. In my hometown, we do not have the diseases. Here, the cases are available, so I afraid I could get disease to other people, so I did not come home. (Migrant worker)***

Q: Thế ở nhà trọ mình thì mình ở 1 mình hay mình ở chung với người khác?

A1: Em thì ở 1 mình.

A2: Em thì ở với bạn.

Q: Bạn là cũng ở trong công ty này à?

A2: dạ vâng, trong công ty này luôn.

Q: Khi phải ở chung như vậy ấy thì kiểu như mình có đảm bảo thực hiện được cái biện pháp ví dụ như giãn cách khoảng cách giữa 2 người không? Đợt trước thì khuyến cáo là 2m xong bây giờ gần đây là 1m thì mình có đảm bảo được các biện pháp đấy không?

A1: Về cơ bản thì vẫn đảm bảo được.

….

IDI-2- Migrant workers-Male-31 years old

….

Q: Bất cứ khi nào gặp vấn đề về sức khỏe thì mình có thể xuống phòng y tế. mình có phải chuyển đi tuyến trên nhiều không anh ?

A: Đúng rồi. từ khi anh vào đây thì chưa thấy, chỉ có họ cảm cúm mệt mỏi hay đa số là các công nhân nữ họ đến tháng thì đau bụng. còn về cái chuyển tuyến trên thì chắc sẽ có nhưng từ khi anh vào đây thì chưa có trường hợp đấy.

Q: Ngoài vấn đề đấy thì còn vấn đề nào khác mà công nhân hay gặp không?

A: Hiện tại thì các vấn đề sức khỏe khác ở công ti này thì hiện nay không có vấn đề gì cả.

Q: Y tế mình thì ngoài việc theo dõi chăm sóc sức khỏe của công nhân hiện nay thì hàng ngày y tế của mình còn làm việc gì khác?

A: Thường thì họ sẽ có các cái đào tạo định kì về cái sức khỏe như là mời các bác sĩ chuyên gia của đa khoa Bắc Ninh về nói chuyện về các cái chuyên đề trong các buổi kiểm kê, còn trong cái thời điểm dịch covid phát sinh thì có rất nhiều các hoạt động của bên y tế. cái thứ nhất đấy là họ tuyên truyền ngay từ ban đầu tức là tuyên truyền về các cái bang rôn treo ở nhà máy, loa phát thanh trong xưởng ngoài ra họ cũng tuân thủ các khuyến cáo của bộ y tế như là đo nhiệt độ trước khi vào công ti hay bắt buộc phải đeo khẩu trang và bên cạnh ấy thì có vệ sinh bằng các cái dung dịch sát khuẩn cloramin b với cả phun khử trùng toàn bộ nhà máy định kì. Ngoài ra còn có người nước ngoài nữa, nên là hết thời gian nhập cảnh ở Việt Nam theo quy định của bộ y tế thì buộc phải khai báo y tế, đo nhiệt độ tiến hành đeo khổ trang trong suốt quat trình làm việc hết 14 ngày.

Q: Như vậy là mình cũng có 1 số vấn đề về phòng chống covid trong cái giai đoạn hiện nay, có tuyên truyền thì cái tuyên truyền này mình chủ yếu dùng tờ rơi hay qua âm thanh.

*A: Qua hệ thống âm thanh, tức là mỗi xưởng họ sẽ có 1 cái hệ thống tivi ở phòng thay đồ ở trên đấy sẽ đọc cái khuyến cáo những vấn đề nên hay không nên về covid, phát sinh triệu chứng như vậy hay thế nào cần làm gì thì đều có hết. Nhìn chung thì mình thấy công tác phòng chống dịch chính phủ đang làm rất tốt. Mình cũng xem tivi hàng ngày, cập nhật thông tin về số ca mắc và tử vong trên thế giới. Các thông tin này cũng giúp mình cập nhật, tin tưởng và thực hiện theo các khuyến cáo và hướng dẫn phòng dịch của chính phủ. (**→ Government doing very well. I watch TV news every day, where the updated information on new cases and deaths every day in the world. This information helped me to trust and follow the government action and guidance (Migrant worker)***

Q: Do nhiệt độ hiện nay thì tiến hàng đo những lúc nào?

*A: Thì hiện nay công ti tiến hành đo 100% đối với toàn bộ công nhân viên trước khi vào nhà máy và áp dụng cho tất cả, kể cả những người cung cấp hàng cho công ti thì phải đeo khẩu trang và đo nhiệt độ.**….***

IDI-1-WU representative-Female-45 years old

….

Q: Nhận xét về cái việc tuân thủ thực hiện các biệp pháp phòng chống dịch bệnh thì người dân trên địa bàn nói chung và công nhân từ đeo khẩu trang, sát khuẩn đến việc giữ khoảng cách thì mọi người thực hiện thế nào?

*A: Thực ra thì các cơ quan chấp hành nghiêm rồi, ở cộng đồng thì ý thức đeo khẩu trang rất tốt nhưng cái giãn cách 2m thì không được, ví dụ như ở chợ mà ra chợ thì không thể cách được như thế, mọi người không tụ tập không tổ chức ăn uống, công nhân ý thức chấp hành đợt 1 ý thức tốt hơn, đợt 2 thì có vấn đề hơn, được phát khẩu trang nhưng vẫn không đeo, hỏi thì bảo quên, đợt 1 thì đường rất vắng và thực hiện tốt hơn. Đấy là theo cá nhân chị nhận định như thế thôi. Bọn chị cũng chỉ nhắc nhở nhẹ nhàng thôi. (**→ However, in phrase II, their practices were not quite good, they seemed to ignore, did not wear face-masks or forget to wear face-masks even the factory gave face-masks to them. It was as my opinion and observation in community. And I am a member of women union, so I just gave them a gentle remind to wear face-mask or comply with the guidance of COVID-19 prevention and control (WU representative)***

Q: Trong đợt covid vừa rồi thì chỉ thấy là với bên phụ nữ thì có những thuận lợi và khó khăn gì?

A: Nói đến thuận lợi thì các chị có thể là số lượng hội viên đông. Khó khăn thì không phải tất cả chị em đều có nhận thức tốt, cũng có 1 số không cập nhật thông tin trên các trang mạng xã hội, đa dạng tuổi đa dạng trình độ, cái nhận thức nó không đồng đều.

Q: Mình có biện pháp khắc phục gì không?

A: không có covid thì chị em có thể tuyên truyền bằng các hội nghị nghe viết cũng dễ hơn nhắn trên điện thoại.

Q: Nhắn trên zalo thì mình nhắn những thông tin gì?

A: Những thông tin mà các ban chỉ đạo tình hình do bộ y tế cập nhật số người mắc địa điểm mắc hay biểu hiện bệnh là bọn chị chia sẻ hết.

Q: Mình có trao đổi với nhau trường hợp nào đang cần theo dõi hoặc là f1, f2 không?

A: Có chứ f1, f2 có bao nhiêu trường hợp là người của địa phương liên quan như thế nào thì các chị lại thông tin đến các thôn, các chị dưới thôn sẽ nắm bắt rõ hơn.

….

Q: Mình có mối liên hệ nào với chủ nhà trọ để thông qua đấy mình kết nối với công nhân không?

A: Ở đây thì bọn chị có hẳn 1 câu lạc bộ nữ chủ nhà trọ, chính các chị đấy sẽ tuyên truyền cho các bạn luôn, gặp là nhắc nhở ngay.

Q: công nhân bị ảnh hưởng giảm giờ làm thì mình có hỗ trợ gì không?

A: Riêng cái hỗ trợ là các chị giảm giá phòng trọ cho, các chị trong câu lạc bộ bảo nhau thôi, giúp các em giảm tiền phòng trọ xuống.

Q: Chị có biết giảm bao nhiêu không?

A: Chị cũng không rõ đâu hình như là giảm 1 vài trăm gì đấy với bình thường. còn có những trường hợp đặc biệt các chị còn không lấy tiền cơ nhưng ít trường hợp lắm.

Q: Ngoài ra nữa thì chị có các hoạt động hỗ trợ gì thêm nữa không?

A: Có 1 số người người ta không muốn thông tin của mình của mình bị biết thì có bạn ủng hộ hơn 1000 cái khẩu trang, đến mua đồ nhỏ thôi bạn cũng tặng nhiều khẩu trang, chị cũng muốn chia sẻ gương tốt nhưng bạn không muốn chia sẻ thông tin cá nhân. Các chỉ cũng ủng hộ ban chỉ đạo của xã của huyện, tổng trị giá là gần 8 triệu. các chị quyên góp trên tinh thần tự nguyện không phải đi thu mà mọi người đem đến. (→ ***There are some people who donate but do not want to share their personal information. Someone supported more than 1,000 face-masks, I told them that I want to share a good example but they did not want to share their personal information. We also support the COVID-19 commune committee, the total is nearly 8 million VND [about USD 346]. We donated in the voluntarily spirit, someone in community just come and brought their donations (WU representative)***

….

IDI-2-WU representative-Female-42 years old

….

Q: Nhóm công nhân này bị thiếu bị các cái vấn đề về cắt giảm giờ làm rồi mất việc ấy thì có nhận được sự hỗ trợ nào từ phía các cơ quan đoàn thể và đặc biệt từ hội phụ nữ không?

A: Khi mà các cái chính sách hỗ trợ của đại dịch covid thì ban chỉ đạo xã cũng đã họp với các ban ngành đoàn thể thông báo rà soát tới từng địa phương, từng các cấp hội và từng các chi hội để thuộc các đối tượng nào thì người lao động đến kê khai trực tiếp và những đối tượng ấy được nhận những hỗ trợ đúng đối tượng còn đối với các doanh nghiệp nếu như mà nghỉ đúng theo thì được hưởng chế độ doanh nghiệp thì cũng đề nghị các hội viên và hộ lao động người ta đến trực tiếp doanh nghiệp. Nhìn chung là công nhân và người lao động cũng rất phấn khởi thứ nhất là cái hỗ trợ cái đại dịch covid đợt 1, những đối tượng khó khăn khi người ta đến khai báo thông tin thì cũng chưa nắm bắt được nhưng khi đợt 2 thì người dân nguopwfi lao động người ta cởi mở hơn.

Q: Như vậy là mình hỗ trợ được cho bao nhiêu người và trong đấy thì có bao nhiêu công nhân?

A: Tức là đối với cái công nhân mà lao động mất việc làm ở các cái doanh nghiệp thì do các doanh nghiệp xác định và được hưởng mức độ hỗ trợ tại ủy ban nhân dân xã cái này thì cũng hạn chế còn đối với những người lao động tự do và những người lao động không nằm trong khu công nghiệp thì theo những quy định định mức của địa phương mà người ta cũng nhận được ở bên xã Ninh Phúc khoảng hơn 223 hộ, người ta đã rà soát và mang đơn lên giải quyết.

Q: Như vậy thì hộ dân sẽ được xã giải quyết còn công nhân sẽ do bên công ti hỗ trợ.

A: Cái nào thuộc thẩm quyền của xã thì sẽ gửi còn không thuộc thẩm quyền thì

Q: Công nhân mà đủ điều kiện thì có bao nhiêu công nhân đủ điều kiện hỗ trợ trên xã?

A: Cái đấy đợt 1 thì ít nhưng mà đợt 2 thì cũng chưa năm bắt được cụ thể số lượng.

Q: Đợt 1 được bao nhiêu người?

A: 7 người.

Q: Thế thì mình được hỗ trợ những gì?

A: Được hỗ trợ bằng tiền.

Q: Bao nhiêu tiền?

A: Hỗ trợ định mức thì hỗ trợ 1 triệu/ 1 tháng và được hỗ trợ 3 tháng theo định mức thì từ ngày 1/4.

Q: Thế nhóm công nhân được hỗ trợ 3 tháng này thì các tiêu chuẩn như thế nào, chị có biết không?

A: Cái tiêu chuẩn cụ thể thì có lẽ chị cũng chưa nắm bắt được cụ thể chỉ có rà soát các đối tượng mới biết được cụ thể và chặt chẽ.

Q: Tức là khi mình rà soát thì công nhân sẽ nộp đơn lên. Thế thì có nhiều người nộp đơn không?

A: Có chứ. Khi ngay đợt 1 là người dân người ta cũng không hiểu mấy, tất cả người lao động và công nhân khi người ta nghe thấy cái hỗ trợ của thủ tướng chính phủ, tất cả người lao động đều nộp đơn hết nhưng sau khi nộp đơn về xã thì xã cũng tổ chức hội nghị theo cái chỉ đạo của cấp trên và người ta công bố là đối tượng nào được hưởng thì thật ra là khi mà rà soát đến các đối tượng được hưởng đều rất khó khăn. ***(→ The commune local authorities also organized the meeting to announce the government support package. Most people were applying for the support at the beginning. However, very few (7 people out of 223) got the support due to very strict criteria for being beneficiary people (WU representative).***

Q: Ưu tiên các đối tượng khó khăn nhiều nhất đúng không?

A: Đúng rồi.

Q: Hội phụ nữ mình có tham gia các hoạt động hỗ trợ thêm như là ở đây có hoạt động gì liên quan tới từ thiện mà hội mình được tham gia không?

A: Xã Ninh Phúc thì chỉ có ban công tác mặt trận thì người ta mới có cái hỗ trợ ở trên còn hội phụ nữ chỉ tham mưa cùng thôi, tham gia cùng chứ cũng không có kinh phí để tổ chức.

Q: Vâng, mình tham gia cùng mấy đợt?

A: Được 2 đợt.

Q: Phát động được bao nhiêu người dân?

A: 2 đợt phát gạo.

Q: Thế là mỗi người dân thì được bao nhiêu.

A: được 1 túi 5 cân 10 cân theo từng đối tượng

….

IDI-2-Factory health worker-Female-35 years old

….

Q: Khẩu trang thì mình bắt người ta đeo luôn từ 1/2 hay như thế nào?

A: Khẩu trang thì bắt đầu từ 1/2 là bắt buộc ạ, vâng 100% và bên phía công đoàn của công ty có hỗ trợ mỗi 1 cán bộ công nhân viên trong công ty là 4 khẩu trang vải.

Q1: À, tức là hỗ trợ cố định luôn?

A: Vâng, khẩu trang vải, đấy là cán bộ công nhân viên làm tại MCNEX còn đối với tất cả công nhân mà gọi là công nhân mới đấy ạ, là cũng được phát khẩu trang khi họ bắt đầu vào công ty làm việc ngày đầu tiên.

Q: Tức là mình đứng về phương diện y tế đúng không, tức là mình đo nhiệt độ các thứ vân vân.., còn kiểm tra rồi các thanh tra của cấp trên và ban ngành liên quan thì như thế nào?

A: Đối với các cái cơ quan ban ngành thì bọn em sẽ được hướng dẫn là từ xa, đối với riêng Ninh Bình thì công ty em thuộc diện quản lý của trung tâm kiểm soát dịch bệnh Ninh Bình và đơn vị gần nhất là TYT xã ở phường Thanh Sơn là họ có vào kiểm tra hướng dẫn và đưa các thông tư, thông báo của BYT về hướng dẫn các biện pháp phòng chống covid như thế nào để cho công ty hoạt động theo quy định của nhà nước. ***(→ My company is under the management of Ninh Binh provincial CDC and Thanh Son commune health center. During the pandemic, health workers came and guided to follow the instructions from the Ministry of Health on COVID-19 prevention and control measures. So that, the company could understand and comply with the national regulations on COVID-19 prevention and control. (Factory health worker)…***

Đối với người nước ngoài khi đến công ty làm việc thì không có người từ năm 2019 trước khi xảy ra covid thì nhà em có các supporter có nghĩa là các chuyên gia của công ty tổng sang Việt Nam để hỗ trợ, nhưng bắt đầu từ khi covid tại Việt Nam thì không có những trường hợp đấy, không sang nữa, sẽ không cho nhập cảnh nữa. Và tất cả nhưng quản lý ở đây sẽ tự hỗ trợ.

Q: Tức là tóm lại là gần như không có người bên kia sang hỗ trợ nữa mà chỉ có điều khiển từ xa hoặc họp hành từ xa đúng không?

A: Đúng rồi ạ, họp trực tuyến online. Còn đối với những Hàn sang công ty trong tháng 1 thì có kiểm tra, điều tra và theo dõi nhiệt độ và cho người ta cái phiếu kiểm tra lịch trình trước khi sang Việt Nam thì họ đã đi những đâu và khi sang Việt Nam đến sân bay Nội Bài đến MCNEX thì họ đã đi những đâu, thời gian là khoảng bao lâu.

Q: Có 1 biện pháp hay được nói đến là biện pháp phun khử khuẩn thì có làm không?

A: Cũng bắt đầu phun từ 1/2.

Q: Mình phun hàng tuần, hàng tháng hay như thế nào?

A: Nhà em đối với khu vực nhà ăn, nhà vệ sinh thì phun 1 tuần/lần vào ngày chủ nhật khi công nhân nghỉ, còn các khu vực khác thì sẽ là 2 tuần/lần đối với khu vực bên ngoài. Còn sàn nhà và tất cả các khu vực sàn nhà văn phòng, tất cả hành lang và sàn nhà nhà vệ sinh, cầu thang, hành lang đều được khử khuẩn và lau bằng cloramin B hằng ngày luôn, ngày lau 2 lần vào đầu buổi sáng và đầu buổi chiều.

….

IDI-1-YU representative-Male-32 years old

….

Q: Anh vừa nói triển khai về hoạt động truyền thông, còn đây là hỗ trợ. Thế thì anh có thể nói rõ hơn về việc phối hợp với các ban ngành khác như công an, trạm y tế,… phối hợp như thế nào?

A: Cái việc phối hợp đó thì theo đúng chỉ đạo về hoạt động vệ sinh môi trường, công tác phòng chống dịch này cũng như là phối hợp phát tặng quà cho các đối tượng COVID ý. Và đặc biệt công tác chốt dịch thì chúng tôi thực hiện công tác trực chốt dịch nữa.

Q: Vậy công tác phối hợp có gặp nhiều khó khăn không?

A: Công tác phối hợp thì phải nói là rất nhiều thuận lợi, hầu như không có khó khăn gì. Nếu có khó khăn thì cũng chỉ là các bạn trực chốt nhắc nhở các bạn công nhân đi làm không đeo khẩu trang chẳng hạn. Thế thì các bạn trực chốt đã yêu cầu nhắc nhở nhưng vẫn có người không phối hợp thực hiện. Thế thì việc đó chúng tôi lại phải có công an can thiệp.

Q: Các ban ngành đoàn thể thường xuyên tương tác. Thế còn công ty có sự phối hợp gì không?

A: Các công ty trong phòng chống dịch thì các công ty có phối hợp tuyên truyền với công đoàn trong đó. Thế thì chúng tôi đã có nhiều năm phối kết hợp tuyên truyền, vận động công ty, công đoàn công ty phòng chống dịch. Hiện có công ty tôi không nhớ tên nhưng trong báo cáo có ghi rõ là chúng tôi có phát gạo, quần áo cho công nhân trên địa bàn, việc rất là thiết thực.

Q: Có công ty nào không phối hợp không?

A: Thực ra các công ty thì họ cũng có những hoạt động riêng, họ cũng thực hiện các công tác phòng chống dịch. Thực sự ra tổ chức đoàn cấp xã chỉ phối hợp với 1 số công ty trong địa bàn mà mình đã giao lưu từ trước thì mình có thể phối hợp tuyên truyền được. Nhưng cũng có những công họ có đặc thù riêng, mình không thể phối hợp được.

Q: Thế là anh thấy hoạt động phối kết hợp với một số công ty thì mình có tổ chức việc phát gạo cho số công nhân nghỉ việc. Vậy số công nhân nghỉ việc, giảm giờ làm nhiều không?

A: Theo mình biết thì số công nhân đó trên địa bàn so với tổng số công nhân thì không có nhiều. Nó rơi vào khoản độ 2% công nhân trên địa bàn thôi. Theo như theo dõi công quản lý thì trên địa bàn có khoảng 20.000 công nhân thì số bị khoảng 1-2% số đó thôi chứ cũng không nhiều vì cao điểm tháng 4.

Q: Thế là với 2% đó là mình có phát gạo, phát quần áo đó. Mình có được sự tài trợ của tổ chức nào không?

A: Gạo và quần áo thì các công đoàn công ty phối hợp.

Q: Tức là đến từ các công ty? Thế ngoài ra còn nguồn nào không?

A: Ngoài công ty ra thì còn có rất nhiều mạnh thường quân. Cái đấy họ làm trực tiếp. Nếu mình tuyên truyền vận động thì họ sẽ ủng hộ. Còn họ thường sẽ ủng hộ trực tiếp công nhân trên địa bàn.

Q: Cái việc tuyên truyền chủ nhà trọ giảm tiền phòng có thực hiện được không?

A: Có, cũng có một số nhà trọ vận động cũng giảm. Tuy nhiên vì lợi ích thì cũng có nhiều nhà trọ không giảm.

Q: Giảm bao nhiêu phần trăm?

A: Cái này thì rất khó thống kê vì các nhà có các nhà trọ mình cũng không thống kê được hết. Nhưng theo một số tuyên truyền thì chúng tôi biết được có 14 chủ nhà trọ họ có giảm tiền. Ví dụ đang cho thuê các bạn 700-800 thì được giảm còn 300-400. Giảm một nửa đó. Còn có các nhà cho miễn tiền nhà 1 tháng trong khoảng thời gian đó. (→ ***This is very difficult because I cannot list all the rental houses. But according to some information, we know that there are 14 landlords have reduced the renting room for workers. For example, they reduced from 700-800,000 VND to 300-400,000 VND. Reduced by half. Other landlords, they offered one month free of charge for renting room during that peak of COVID-19. (YU representative)***

….
